# Reply to the Letter to the editor in response to the Position statement and best practice recommendations on the imaging use of ultrasound from the European Society of Radiology ultrasound subcommittee

**DOI:** 10.1186/s13244-021-01003-8

**Published:** 2021-05-21

**Authors:** Adrian P. Brady, Dirk-André Clevert, Paul S. Sidhu, on behalf of the European Society of Radiology ultrasound subcommittee

**Affiliations:** 1 Radiology Department, Mercy University Hospital, Grenville Place, Cork, T12 WE28 Ireland; 2 University College Cork, Cork, Ireland; 3 Department of Clinical Radiology, Interdisciplinary Ultrasound-Center, Munich University Hospital, Munich, Germany; 4 Department of Radiology, King’s College Hospital, London, UK; 5 Am Gestade 1, 1010 Vienna , Austria

Dear Editor in Chief,

We welcome the letter's authors' interest in our paper “Position statement and best practice recommendations on the imaging use of ultrasound from the European Society of Radiology ultrasound subcommittee”, published in Insights into Imaging in November 2020 [1].

The Ultrasound subcommittee of the European Society of Radiology endeavours to focus in its publications on matters of broad interest across our member countries [2–5]. Our principal goal is to promote appropriate use of ultrasound as an imaging modality, and to define standards for such use, in the best interests of patients. Matters of licensing, registration, qualifications and regulation of who performs ultrasound imaging are specific to each country, and vary widely across Europe. Such matters, while of importance within each individual country, are beyond the scope of ESR publications on standards.

We re-affirm that ultrasound should be performed by fully-trained, competent individuals, who adhere to formal standards of required training, education and practice (as outlined in our position paper), and who abide by their relevant country’s regulatory regime.

Yours sincerely.

## Data Availability

Not applicable.
